# Survival of microorganisms during two-year exposure in outer space near the ISS

**DOI:** 10.1038/s41598-023-49525-z

**Published:** 2024-01-03

**Authors:** Elena A. Deshevaya, Svetlana V. Fialkina, Elena V. Shubralova, Oleg S. Tsygankov, Natalia M. Khamidullina, Leonid M. Vasilyak, Vladimir Ya. Pecherkin, Viktoria A. Shcherbakova, Andrey M. Nosovsky, Оleg I. Orlov

**Affiliations:** 1 State Scientific Center of the Russian Federation - Institute of Biomedical Problems of the Russian Academy of Sciences, Moscow, Russia; 2 Joint Stock Company «Central Research Institute for Machine Building», Moscow Region, Russia; 3 Rocket and Space Public Corporation Energia, Moscow Region, Russia; 4 Corporation «Lavochkin Scientific and Production Association», Moscow Region, Russia; 5 Joint Institute for High Temperatures of the Russian Academy of Sciences, Moscow, Russia; 6 Pushchino Scientific Center for Biological Research of the Russian Academy of Sciences, Skryabin Institute of Biochemistry and Physiology of Microorganisms, Moscow Region, Russia

**Keywords:** Biochemistry, Biophysics

## Abstract

Results of an experiment named "Test" on survival and variability of microorganisms in open space near the International Space Station are presented. It was found after two-years exposure, spore-forming bacteria of the species *Bacillus subtilis*, fungi of the species *Aureobasidium pullulans* and archaea of the species **Methanosarcina mazei S-6**^**T**^, deposited on cotton wool, are able to survive, and their numbers decreased equally, regardless of whether the microorganisms belong to different taxonomic groups. The main factors for the long-term survival could be the result of their dehydration and partial lyophilization in the vacuum of near-Earth space. For the first time, after being in outer space, cyst-like cells of the archaea strain *M. mazei* **S-6**^**T**^ and a 14-day delay in their growth were detected when cultured on a nutrient medium compared to the ground-based control strain. In 30% of fungi species strains *A. pullulans*, isolated after a two-year stay in outer space, the resistance to **γ—**radiation increased compared to the control strain. It was found that the reaction to the action of various concentrations of hydrogen peroxide and 1% chlorine in the surviving strains of the fungus *A. pullulans* on the ISS is less pronounced than in the control strain.

## Introduction

A lot of experiments carried out in the space vacuum near the International Space Station (ISS) have been aimed at studying the potential of biological objects and structures to adapt and survive in specific space conditions, which will answer the main technogenic panspermia question of to the possibility of their transfer to other planets using technical facilities. The outer surface of the ISS, which has been in orbit for more than 20 years, is an ideal experimental platform for studying the survival of microorganisms in outer space. The experiments "Exposure-E"^1–4^, "Exposure-R"^5–7^, "Biorisk"^8,9^, "Tanpopo"^10^ have been conducted on the outer surface of ISS by the leading space agencies to determine the resistance of various biological objects and microorganisms to damaging factors of near-Earth space. With up to six month experiments, it was shown that biological objects and microorganisms are able to survive under various damaging factors. However, in these experiments, the effect of a limited number of outer space physical factors was tested. For example, in the "Exposure-R" experiment^5,6^, the effect of cosmic UV radiation was studied and simulated in a control terrestrial experiment, which were concentrated into special polymer bags before being placed in a metal three-layer track, thereby protecting biological objects from the vacuum existing in space. In the experiment "Biorisk"^8,9^, the resistance of microorganisms to the effects of the parameters of the cosmic vacuum was studied, and the metal body protected microorganisms from the action of UV radiation. In the "Tanpopo" experiment^10^, microorganisms were placed in special tracks that were covered with glass. So, in the experiments carried out, the exposed biological objects were isolated from the action of other factors in outer space by special barriers, such as metal casing, membranes, filters, glasses, which did not allow investigate the effect of the full range of space physical factors. During the operation of the ISS, it was discovered that the outer surface of the modules was contaminated with "space dust" caused by various factors. Determination of physicochemical properties and investigation for the detection of biological structures and viable microorganisms in this "space dust" was one of the main scientific tasks. To do this, starting in 2010, the first stage of the experiment "Test" was conducted on the ISS. In this experiment, for the first time, the possibility of regular sampling of "space dust" in its natural state from the outer surface of the ISS and delivering these samples to Earth for laboratory research was realized. A device has been developed that allows astronauts to select "space dust" and hermetically pack it into containers for transportation to Earth^11^. The device ensured the sterility of the sampled dust particles from the ISS surface, up to their transfer to the laboratory. The results of biological studies of the first stage demonstrated the presence of biological objects in the selected samples of "space dust". The composition of DNA of biological structures and viable units of microorganisms isolated from samples taken from various elements of the outer surface of the ISS was determined^12,13^. Analysis of the data obtained shows that the DNA of archaea, spore bacteria and fungi, as well as viable units of bacteria of the genus *Bacillus* and fungi *A. pullulans* were most often found in these samples.

The identification of single viable strains of microorganisms did not answer the question about duration of their survival in space conditions. In this regard, the second stage of the experiment "Test" was carried out, in which we investigated the time microorganisms viability of during exposure in open space.

The goals of this work were the selection of microorganisms species for exposure and the study of the duration of it survival in open space near the ISS, as well as changes in radiation resistance and the influence of individual stress factors on the fungi strains.

## Results

At the first stage of preparation, laboratory studies were carried out on the survival of microorganisms under the influence of ultraviolet radiation and their persistence on various surfaces with a rapid pressure drop arising from the depressurization of the transport device. The studies were carried out with spore-forming bacteria and fungi previously isolated from the inner and outer surfaces of the ISS, as well as with archaea strains of the All-Russian Collection of Microorganisms (VKM). All strains of microorganisms were exposed to ultraviolet radiation with a wavelength of 253.7 nm at three different fluences: 12 J/m^2^, 24 J/m^2^ and 60 J/m^2^. Modeling of the pressure drop from 1 bar to 3 × 10^–4^ mbar was conducted in a vacuum chamber on different surfaces^14^. The main selection criteria were considered to be the largest number of surviving microorganisms after exposure to the maximum dose of ultraviolet radiation and the largest number of microorganisms preserved on the surface after a pressure drop. As a result of the conducted studies, the following microorganisms were selected for subsequent tests in outer space with an exposure duration of 12 and 24 months—bacterial spores of the *B. subtilis* species isolated from the outer surface of the ISS porthole^15^, fungal spores of the *A. pullulans* species, isolated from the outer surface of the radiator of the ISS temperature control system^15^, archaea of the *M. mazei* S-6^T^ species, collection strain VKM B-1636T^16^.

### Post-exposure testing of microbial survivability

**Figure 1:**
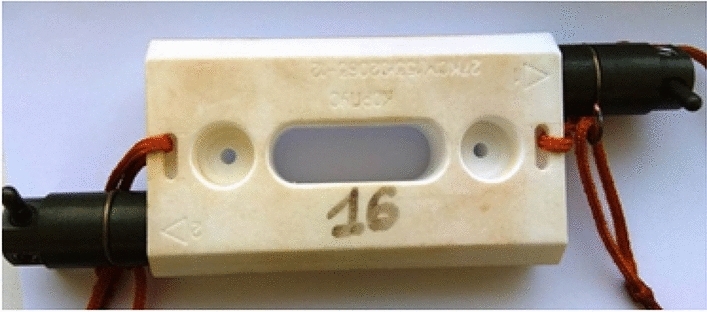
Photo of the “Test” transport module with the rods installed after 2 exposure years.

After the completion of each stage of the exposure (12 and 24 months) on the outside of the ISS, the rods with microorganisms were installed in sealed transport modules "Test" and delivered to the laboratory. A photo of the “Test” transport module is shown in Fig. 1.

**Figure 2:**
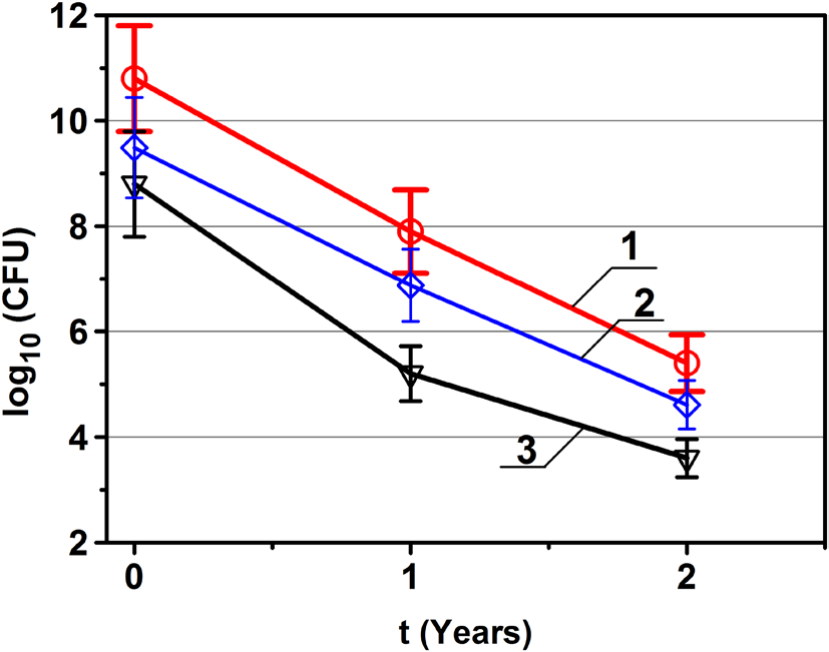
Change in CFU abundance during exposure in open space near the ISS. 1—*Bacillus subtilis*,* 2—Methanosarcina mazei* S-6^T^, 3—*Aureobasidium pullulans.* CFU of archaea corresponds to the cells number determined by the methane production.

The number of surviving spores of bacteria and fungi was determined by the viable colony-forming units (CFU) that grew during surface sowing on solid nutrient media. The number of surviving methanogenic archaea was determined by the methane formation^16^. Figure 2 shows the decrease in the number of viable colony-forming units (CFU) of microorganisms vs. exposure time in open space.

From the above we can see that in the first year the number of CFU bacteria, archaea and fungus decreased by 3 orders of magnitude, in the second year the number of CFU of all microorganisms decreased by another 2 orders of magnitude compared to the first year and by 5 orders of magnitude compared to the initial number.

### Post-exposure studies of archaea *M*. *mazei* S-6^T^

The growth of the archaea population was determined by the methane formation during cultivation on a nutrient medium. As a result, a 14-day delay in the appearance of methane was revealed during cultivation of the *Methanosarcina mazei* S-6^T^ strain after exposure, which was not observed in the ground control strain^16^.

**Figure 3:**
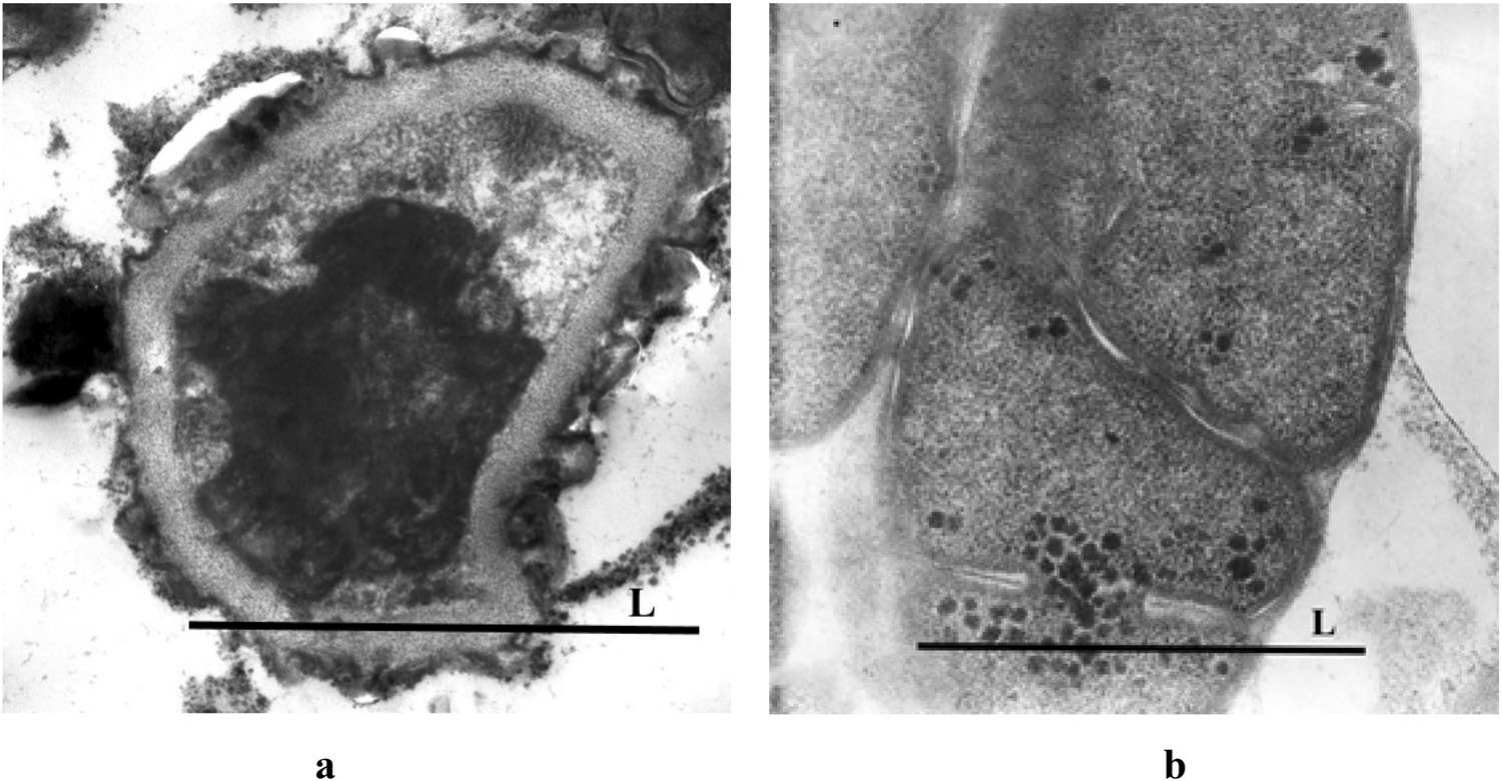
SEM images of *M. mazei* S-6^T^ cells: (**a**) ultra of *Methanosarcina* cell from the isolate obtained after the 24-mos exposure; (**b**) ultrafine of *Methanosarcina* cell control. Scale – L = 1.0 micron.

Electron microscopy studies of cells after 24-month exposure showed an unexpected formation of cyst-like methanosarcin cells in space (Fig. 3a), characterized by the presence of a multilayer thickened cell membrane, which has never been observed in strains of this species on Earth, as well as in the control strain in our experiments (Fig. 3b)^16^.

### Post-exposure studies of fungus *A*. *pullulans*

This fungus belongs to radiotrophic fungi that survive in conditions of increased radiation levels. Therefore, we carried out studies of *A. pullulans* fungi of a strain from the VKM collection (VKM F-1116), a strain previously isolated from the outer surface of the ISS (isolated strain) and individual isolated subcultures of this isolated strain after two years of exposure in open space (subcultures 2.1–2.15) to determine their survival under the influence of γ—radiation, various stress factors such as elevated temperature, various chemical agents.

### γ—radiation resistance

The spores of each strain were exposed to **γ—**radiation generated by the ^60^Co isotope at the GU-200 M facility. The survival results of fungi of the species *A. pullulans* after exposure to various doses of **γ—**radiation are shown in Table 1.

**Table 1:** Survival of spores of various strains of *Aureobasidium pullulans* after exposure to various doses of gamma radiation.

Strains/subcultures	Initial concentration (100%), (log CFU/ml)	Dose of γ-radiation, kGy				
		1	2.5	5	10	15
Control strain VKM F-1116	6.51 ± 0.58	5.83 ± 0.39/21.3%	3.59 ± 0.61/0.12%	1.26 ± 0.6/0.006%	0	0
Isolated strain	6.88 ± 0.36	6.71 ± 0.41/67.1%	5.08 ± 0.74/0.165%	3.89 ± 0.58/0.1%	0	0
Subculture 2.1	6.79 ± 0.33	6.56 ± 0.55/59%	5.00 ± 0.58/1.64%	4.73 ± 0.48/0.89%	2.95 ± 0.31/0.014%	1.00 ± 0.46/0.0002%
Subculture 2.2	6.64 ± 0.38	6.54 ± 0.61/79%	5.41 ± 0.62/5.9%	4.91 ± 0.7/1.84%	1.00 ± 0.29/0.0002%	0
Subculture 2.3	6.96 ± 0.42	6.87 ± 0.42/82.2%	5.20 ± 0.33/1.7%	3.67 ± 0.55/0.05%	0	0
Subculture 2.4	6.91 ± 0.39	6.26 ± 0.37/21.9%	–	3.49 ± 0.64/0.038%	0	0
Subculture 2.5	6.82 ± 0.59	5.94 ± 0.71/13.3%	–	2.85 ± 0.31/0.01%	0	0
Subculture 2.6	6.96 ± 0.48	6.62 ± 0.51/45.7%	4.92 ± 0.47/0.9%	3.36 ± 0.43/0.025%	0	0
Subculture 2.7	6.86 ± 0.41	6.18 ± 0.43/20.5%	–	3.15 ± 0.7/0.02%	0	0
Subculture 2.8	6.74 ± 0.56	6.32 ± 0.62/38.2%	–	3.00 ± 0.41/0.018%	0	0
Subculture 2.9	6.68 ± 0.33	6.78 ± 0.83/62.5%		4.05 ± 0.35/0.23%	1.08 ± 0.52/0.00025%	0
Subculture 2.10	7.15 ± 0.68	5.01 ± 0.36/7.3%	–	2.61 ± 0.49/0.0029%	0	0
Subculture 2.11	6.98 ± 0.47	5.90 ± 0.49/8.3%	–	3.30 ± 0.53/0.02%	0	0
Subculture 2.14	6.87 ± 0.52	6.26 ± 0.65/24.3%	–	3.92 ± 037/0.1%	0	0
Subculture 2.15	6.84 ± 0.63	6.75 ± 0.49/40.6%	–	4.24 ± 0.58/0.25%	1.15 ± 0.42/0.0002%	0

– not tested.

From the data above, the number of surviving CFU units of the isolated strain is higher than that of the control strain BKM F-1116. Moreover, the difference of surviving CFU units increases with increasing **γ—**radiation dose. Single CFU subcultures 2.1, 2.2, 2.9, 2.15 survived at an irradiation dose of 10 kGy, which is approximately 30% of the studied subcultures. Single CFU subcultures 2.1 survived at an irradiation dose of 15 kGy. The reliability of the data obtained is P ≤ 0.05.

Thus, as a result of the research, it was shown that the radiation resistance to **γ—**radiation of the strain isolated from the outer surface of the ISS is higher than that of the control terrestrial strain VKM F-1116, and 30% of isolated strain subcultures after 2 years of exposure showed increased radiation resistance compared to the isolated strain.

### Stress factors effect on the fungal strains

It is known that microorganisms that have survived under the action of extreme factors are able to acquire resistance to the effects of other adverse physical or chemical factors^17^. Therefore, we carried out studies to determine the reaction of strains of the fungus *A. pullulans*, to the effects of elevated temperatures. During these studies, the spores of various strains of the fungus were kept at a given temperature for 15 min. The results of the studies are shown in Table 2.

**Table 2:** Growth of spores of various strains of *Aureobasidium pullulans* after 15 min of exposure to different temperatures.

	Temperature			
	37 °C	45 °C	65 °C	85 °C
Control strain VKM F-1116	+	+	–	–
Isolated strain	+	+	–	–
Subculture 2.1	+	+	–	–
Subculture 2.6	+	+	–	–

+ growth, – absence of growth.

Table 2 shows that the growth of all strains is observed at temperatures of 37 °C and 45 °C. There were no differences in the reaction of the control terrestrial strain VKM F-1116, the isolated strain and isolated strain subcultures at all values of the studied temperatures.

For exposure to chemical factors, 1% and 3% solutions of hydrogen peroxide and the disinfectant solution “Chlorapine” with a chlorine content of 1% were selected. The qualitative reaction of the action on strains and subcultures of hydrogen peroxide and chlorine was determined by the zone of fungal growth retardation when applying paper discs wetted with solutions of hydrogen peroxide or chlorine to the surface of the spores of each strain placed on the nutrient medium. The results of the studies are shown in Table 3.

**Table 3:** Zone of *Aureobasidium pullulans* retardation due to contact with hydrogen peroxide and chlorine, (mm).

	Hydrogen peroxide		1% chlorine
	1%	3%	
Control strain VKM F-1116	1	10 ± 1.5	30 ± 2
Isolated strain	0	4 ± 0.5	21 ± 1.5
Subculture 2.1	0	5 ± 1	22 ± 1.5
Subculture 2.6	0	4 ± 1	22 ± 1.5

From the data presented in Table 3, it can be seen that the growth retardation zones of the isolated strain and isolated strain subcultures are significantly lower than those of the control terrestrial strain VKM F-1116 under the action of all the studied solutions. The growth retardation zones of the isolated strain and isolated strain subcultures after 2 years of exposure are almost the same. The reliability of the data obtained is P ≤ 0.05.

## Discussion

The main physical factors of near-earth space acting on the studied microorganisms are: vacuum, solar radiation, cosmic radiation, temperature changes.

The results of the studies showed that after two years of exposure in outer space near the ISS, all the tested microorganisms representing various domains of life survived. Especially noteworthy are the almost identical levels of reduction in the number of bacteria, fungi and archaea in relation to the initial levels, amounting to about 5 orders of magnitude. With a high probability, it can be argued that such an identical decrease in the number of CFU is due to the influence of the state of the cotton wool on the surface of which they were applied. It can be assumed that the outer fibers of cotton wool could shield the sun ultraviolet radiation, and the microorganisms located on the inner fibers of cotton wool were partially protected from the ultraviolet radiation effects, which increased the likelihood of their survival.

We also examined the effect of cosmic and solar radiation on the survival of spores of bacteria and fungi on the ISS outside. The amount of absorbed dose that microorganisms could receive during two years of exposure was calculated using the licensed COSRAD software package created at the Lomonosov Moscow State University Research Institute of Nuclear Physics^18,19^. The total absorbed dose from electrons and protons of the Earth's radiation belts, protons and heavy charged particles of solar cosmic rays and galactic cosmic rays in the locations of microorganisms near the ISS surface did not exceed 0.51 kGy.

The dose of 0.51 kGy could not significantly affect the survival of the bacterium spores of the genus *Bacillus*, because the doses of ionizing radiation, which causes a tenfold decrease in colony-forming units are 2 and 3 kGy for *B. pumilus* and *B. subtilis*, respectively^20^. Effects of ionizing radiation on microbial cells were considered in detail in several reviews^21,22^. It was found that at room temperature highly radiation resistant bacteria are able to survive radiation doses of 20–30 kGy^23,24^ and up to 100 kGy at temperatures close to 0 °C)^25,26^. In Ref.^27^ it is said that at low temperatures *Cryomyces antarcticus* can survive 117 kGy of γ—radiation. Based on the literature above, radiation resistance of microorganisms can increase at low temperatures. It is known that the radiation resistance of bacteria spores is higher than that of fungi spores^16^. Most fungi are more sensitive to ionizing radiation and are completely inactivated in the dose range from 1 to 10 kGy, however, the limit of their stability is not reliably determined^28^. We have not found literary data about splitting of a fungal spore population by level of radiation tolerance. However, there is evidence for a similar divergent effect of repeated exposure of *Escherichia coli* cells to UV radiation^29^.

This lets us assume that the increased radiation resistance to γ—radiation of subcultures of the fungus *A. pullulans* 2.1, 2.2, 2.9, 2.15 (Table 1) following the 2-year exposure can be explained as the reaction to a specific stress-factor of the cosmic space rather than an adaptive reaction to the extreme factors. To substantiate this assumption, molecular genetic studies of the fungal spores of *A. pullulans* subcultures obtained after two years of exposure on the outside of the ISS are currently being conducted.

Post-exposure studies of the elevated temperatures effects have shown that the growth of all fungi strains is observed at temperatures up to 45 °C, and stops above 65 °C (Table 2). There were no differences in the reaction of the control strain VKM F-1116, the isolated strain and subcultures at all values of the studied temperatures. Thus, after exposure in outer space, fungal spores did not acquire additional resistance to the effects of temperature.

The influence of chemical stress factors, such as hydrogen peroxide and chlorine, slows down the growth of fungi of the isolated strain and subcultures in comparison with the control VKM F-1116 (Table 3). It should be noted that there are no differences in the growth of subculture strains after 2 years of exposure and the isolated strain.

Effects of space temperature and vacuum on microorganisms are peculiar. It is common knowledge that beyond the atmosphere the only way of heat exchange of bio-objects with the environment, exclusive of mass ejection events, is thermal radiation heat transfer^30,31^. At contact, due to conduction the heat flux will go only one way, i.e. from a heated item to the bio-object. To put it differently, heat transfer is fully dependent on conduction of a bio-object holder. Metal conduction is high, whereas the conduction of cloths is low. For example, heat transfer coefficient of cotton is equal to 0.05 Wt/m*K^32^. Therefore, microorganisms on inside the cotton tips were better protected against overheating where temperature was lower owing to the heat reducing effect of voids in the cotton pore structure.

In vacuum near the ISS, dehydration of microorganisms can occur, both at low temperatures during freezing of water and during heating, depending on the periodic location of the ISS in orbit in the shaded or in the solar region. These processes are similar to the processes used in lyophilization technology to prepare microorganisms for long-term storage. It is possible that these processes are the main factors for the long-term survival of microorganisms in a vacuum near the ISS. Freeze drying, a recognized and widely used method of preserving microbial strains in collections, increases the viability of spores and germ cells^33–35^. In our experiment, dehydration by cosmic vacuum and low temperature could possibly lead to partial or complete lyophilization of spores.

Resistance of microorganisms to space vacuum was investigated in experiment Biorisk^28,36^. Data of the experiment demonstrated the ability of microorganisms to stay alive years. In experiment Expose-E^2^, the least number of viable spores was detected on the lower metal track that was best shielded from ultraviolet in comparison with the middle and upper tracks. The authors defer discussion as the data need a painstaking analysis. Most likely we should consider also a thermal vacuum effect on bio-objects dependent on the exposure design and heat conduction property of materials in the immediate vicinity to bio-objects. Therefore, it is highly probable that in the two-year space experiment microorganisms on the upper cotton layer died from UV radiation, whereas cells that had penetrated in the cotton survived. Besides, cotton could lessen the temperature drops providing lower or comparatively constant temperature in subsequent layers. In other words, microorganisms could turn into a specific state and, thus, survive on the cotton layers protected from UV radiation at stable and comparatively low temperatures**.**

For archaea, after two years of exposure, methanosarcin cells were first found to have its cyst-like forms, characterized by the presence of a multilayer thickened cell membrane (Fig. 3b). The thickening of the cell wall may be due to a response to the combined effects of vacuum, temperature and solar radiation. In addition, a 14-day delay in the development of archaea after exposure in outer space was revealed, which may be due to thickening of the cell membrane and requires a separate study.

## Conclusion

1. As a result of the space experiment "Test", the possibility of survival of bacteria spores of species *B. subtilis*, fungi spores of species *A. pullulans* and methane-forming archaea of the species *M. mazei* S-6^T^ deposited on cotton wool fixed on metal rods was proved when exposed for two years in open space near the ISS without the use of additional barriers protecting from the effects of various physical factors of outer space.
2. For the first time, the features of surviving archaea *M. mazei* S-6^T^ strain were discovered. After two years of exposure, *Methanosarcina* cells were found to have its cyst-like forms, characterized by the presence of a multilayer thickened cell membrane. In addition, a 14-day delay in the growth of archaea after exposure in outer space was revealed, which may be due to thickening of the cell membrane.
3. A specific feature of population variability was revealed in 30% of strains of *A. pullulans* species fungi to increase resistance to radiation effect after two years of exposure in open space near the ISS.
4. As a result of laboratory studies, it was shown that the reaction to the effect of different concentrations of hydrogen peroxide and 1% chlorine in the control strain of *A. pullulans* VKM F-1116 is more pronounced than in the strain isolated from the outer surface of the ISS and strains after two years of exposure in outer space.
5. From the analysis of the data obtained, it can be assumed that one of the main factors for the long-term survival of microorganisms in open space near the ISS is the process of their dehydration and partial lyophilization in vacuum.

## Methods

### Selection of strains of microorganisms for exposure in outer space

This stage included the cultivation of spore bacteria, fungi and archaea, UV irradiation of cultivated microorganisms, determination of the number of preserved microorganisms deposited on various surfaces after the rapid pressure drop. These studies were carried out with spore-forming bacteria and fungi previously isolated from the inner and outer surfaces of the ISS, as well as with archaea strains from the All-Russian Collection of Microorganisms.

#### Cultivation of spore bacteria

To obtain bacterial spores of the species *B. pumilus* and *B. subtilis*, the technique described by Newcombe was used^37^. Bacterial spores were cultivated on a nutrient medium—potato agar without glucose. The culture was incubated for 24 h at 37 °C, and then 4–5 days at 20–22 °C in natural light. Subsequently, a control microscopic examination of the test strains was carried out (the number of spore forms was at least 99%), then a suspension of spores of each strain of bacteria was prepared in a phosphate buffer using the McFarland 2 turbidity reference standard, which corresponds to the number of microbial cells 6 × 10^8^/ml, and heated for 15 min at 80 °C for inactivation of vegetative forms. Then, by a series of successive dilutions in a sterile phosphate buffer, the concentration of the suspension of each microorganism was achieved equal to 10^5^–10^6^ cells in 1 ml.

#### Cultivation of spore fungi

The spores of the fungi *Aspergillus sydowi, Aspergillus niger, Penicilliun expansum* were obtained by surface cultivation for 7 days on a nutrient medium of Chapek Dox (HiMedia), and spores of the fungus *A. pullulans* were obtained by surface cultivation on a medium of potato-dextrose agar (HiMedia). For the research, a suspension of spores of each fungus was prepared in a concentration of 10^5^–10^6^ cells in 1 ml.

#### Cultivation of archaea

The cultivation of methane-forming archaea was carried out using the technique of anaerobic cultivation of Hangate^38^. In particular, the basic phosphate-mineral medium (PBBM) of the following composition (g/l) was used for the cultivation of *M. mazei* S-6^T^ strain: NaCl 0.9; MgCl_2_ × 7H_2_O 0.2; CaCl_2_ × 2H_2_O 0.1; K_2_HPO_4_ 2.9; KH_2_PO_4_ 1.5; Na acetate 4.0; rezazurin 0.001; vitamin solution 5.0 ml; a solution of trace elements SL-10 10.0 ml; cysteine-HCl 0.5; Na_2_S × 2H_2_O 0.5^16^. For UV irradiation, archaea strains *Methanosarcina articum* M-2^T^, *M. mazei* S-6^T^ и *Methanosarcina mazei* JL01 were grown in 125 ml glass vials with a working medium volume of 60 ml. Quartz tubes with a volume of 30 ml were used for UV irradiation, which were previously sterilized, vacuumed and closed with butyl rubber stoppers. Archaea strains grown in glass vials were anaerobically transferred to quartz tubes for UV irradiation.

#### UV irradiation

Low-pressure gas-discharge amalgam lamps with a UV radiation power of 256 ± 28 W at a wavelength of 253.7 nm were used to irradiate microorganisms. The lamp was placed in a Class 5 microbiological safety laminar box. The distance from the lamp to the surface of the samples was 50 cm. Suspensions at a concentration of at least 1.0 × 10^6^ CFU/ml were prepared for UV irradiation of bacterial and fungal spores. Then 0.1 ml of suspension of each strain was applied to the surface of sterile Petri dishes and dried for 1 h before UV irradiation. Test samples of fungal and bacterial spores were irradiated in Petri dishes, and archaea strains in sealed quartz tubes. All strains of microorganisms were exposed to ultraviolet radiation at three different fluences: 12 ± 1.3 kJ/m^2^, 24 ± 2.6 kJ/m^2^ and 60 ± 6.6 kJ/m^2^.

After exposure of bacterial and fungal strains to UV radiation, 25 ml of R2A (HiMedia) medium at 40 °C was poured into Petri dishes and then were incubated at 30 °C and 28 °C, respectively.

#### Determination of the number of surviving microorganisms deposited on various surfaces during sudden pressure drop

The studies were conducted with spores of various strains of bacteria and fungi. The spores were deposited on the surface of metal plates and metal rods, on cotton wool wrapped and fixed on the surface of metal rods. To ensure a sharp pressure drop from 1 bar to 3 × 10^7^ bar, a vacuum chamber was used. This technique is described in detail in Ref.^14^.

### Preparation for the exposure on the outside near the ISS

#### Spore bacteria B. subtilis and fungi *A*. *pullulans*

Bacterial and fungal spores were grown on the surface of appropriate nutrient media to obtain isolated colonies according to the methods described above. We selected spores from one colony and increased their biomass by surface cultivation of a continuous lawn. The suspension of bacterial spores (5 ml) with a concentration of at least 1 × 10^9^ CFU/ml and a suspension of fungal spores (5 ml) with a concentration of at least 1 × 10^7^ CFU/ml were prepared.

Suspensions of each strain were collected into a sterile, disposable, sealed, oxygen-free syringe and evenly distributed over the surface of cotton wool attached to the metal rod of the transport module of the "Test" device. Each rod was contaminated with one strain. Next, the rods were dried for 4 h at room temperature. After this, the rods were hermetically installed in individual sterile cells of the transport module of the "Test" device.

#### Archaea *M*. *mazei* S-6^T^

The preparation of methane-producing archaea was carried out according to the method described above. Methanol (2.5 g/L) was used as a substrate for methanogenesis. Solutions of phosphates and a reducing agent (a mixture of cysteine-HCl and Na_2_S × 2H_2_O) were prepared and sterilized separately and added to the mineral medium after sterilization. If necessary, the pH was adjusted with 3N HCl or 3N NaOH. The biomass was grown on PBBM medium in a flow of pure nitrogen in 160 ml glass vials with a working volume of medium 50 ml at 37 °C. Growth was determined by the increase in methane in the gas phase, which was proportional to the amount of biomass formed. CFU was determined by comparing the amount of methane formed with the control. The grown biomass was collected into a sterile disposable, sealed, oxygen-free syringe and evenly distributed over the surface of cotton wool attached to the metal rod of the transport module of the Test” device. Next, the rod, contaminated with archaea, was hermetically installed in an individual sterile cell of the transport module of the "Test" device.

Since the exposure time was 1 year and 2 years, to ensure double repetition, we contaminated 4 rods of the transport module of the “Test” device with each strain.

#### Transportation of microorganisms and their exposure in outer space near the ISS

**Figure 4:**
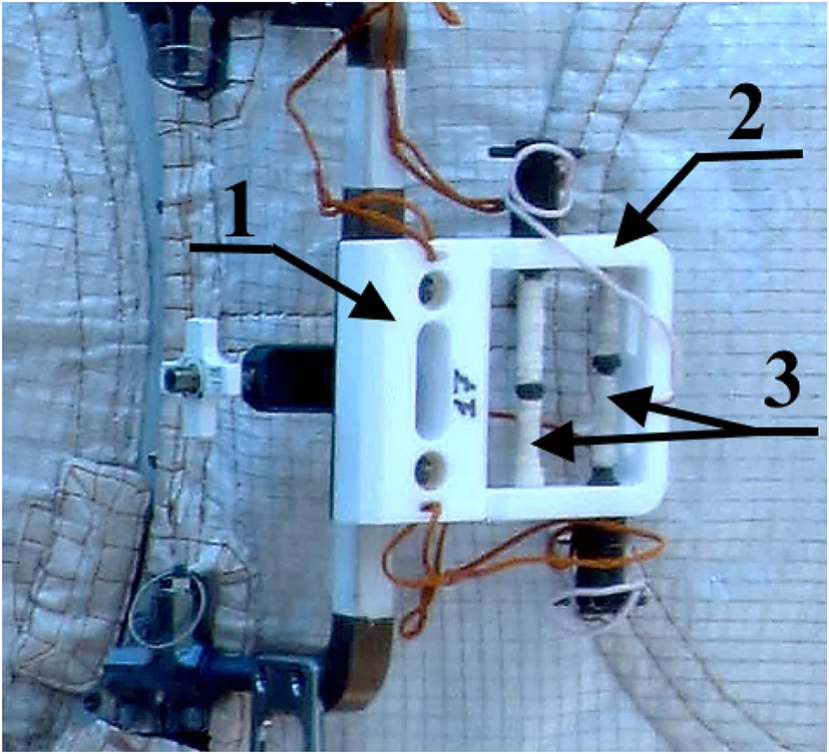
Photo of the “Test” device installed on the ISS outside. 1—transport module; 2—exposure module; 3—rods, wrapped in cotton wool contaminated with microorganisms.

For these purposes, the device "Test" was developed (Figs. 1, 4), intended for the transportation of selected test strains of microorganisms from the earth and their exposure. The device consists of a transport module 1 (Fig. 4), an exposure module 2 (Fig. 4) in which rods with cotton wool contaminated with microorganisms are fixed 3 (Fig. 4). There are threaded holes in the housing of the transport module 1, into which rods 3 are screwed. The design of this part of the device made it possible to maintain the tightness of the delivered microorganisms during transportation. During the spacewalk, the astronaut unscrewed the rod 3 from the transport module and fixed it in a special free hole of the exposure module, as shown in Fig. 4.

**Figure 5:**
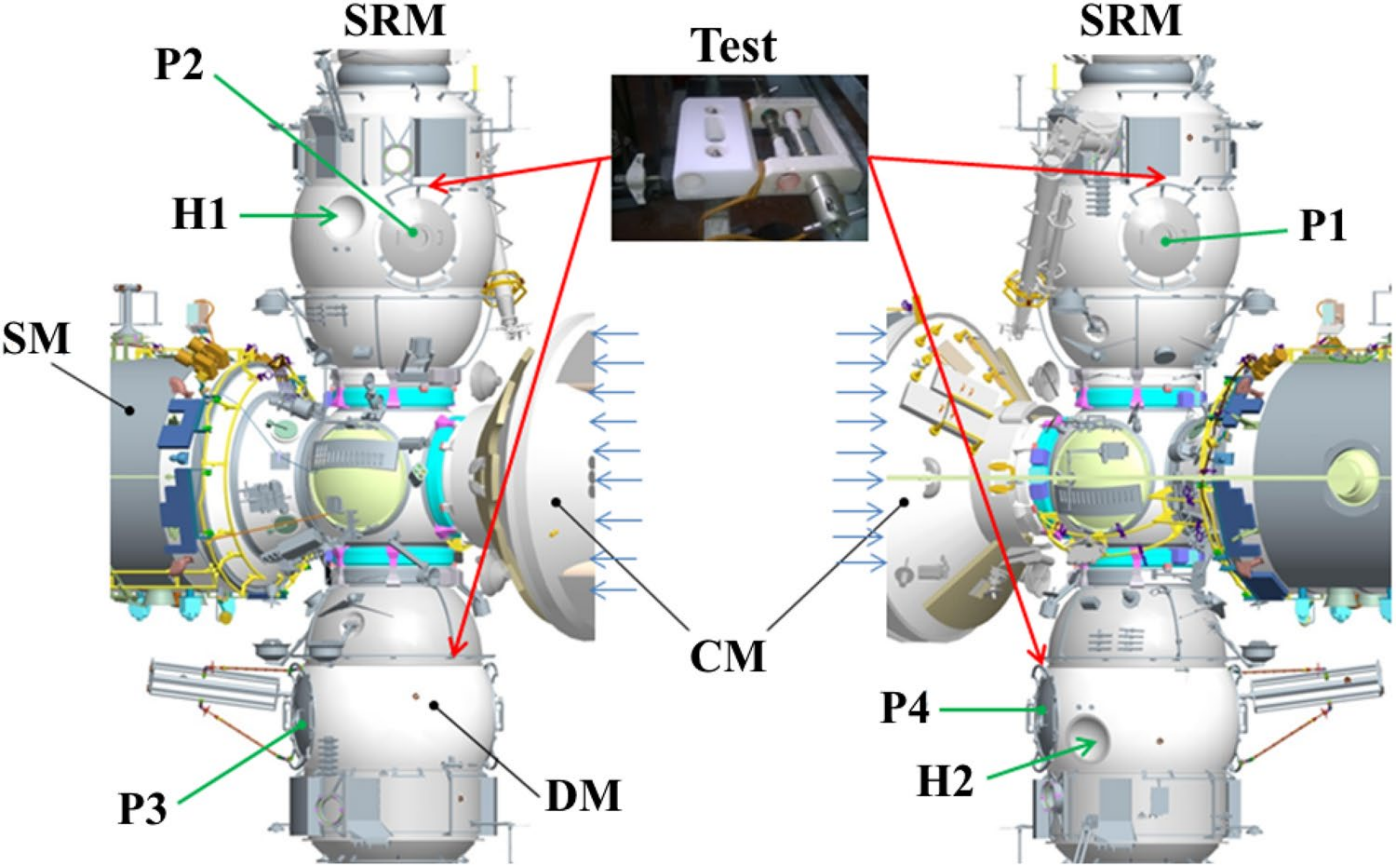
“Test” device installations locations on the outside of the Russian ISS module. Test "Test" device, *SRM* small research module, *CM* cargo module, *DM* docking module, *SM* service module (SM), P1–P4 portholes, H1–H2 hemispheres.

The astronaut placed devices with microorganisms on the outside of the Russian ISS module (Fig. 5). The installation locations of the "Test" devices are indicated by red arrows.

**Figure 6:**
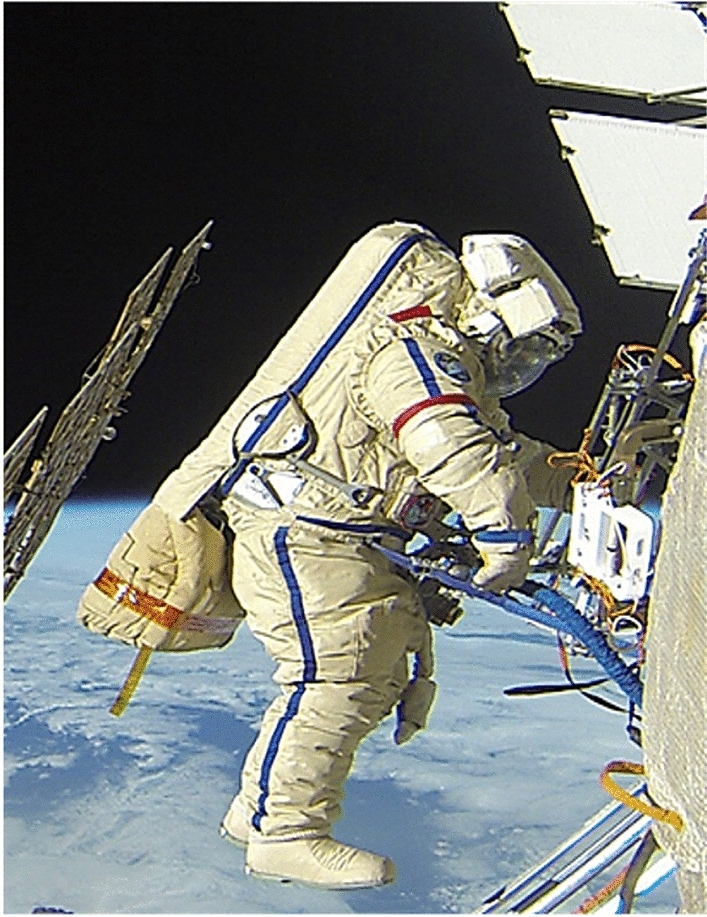
Photo of the installation process of the "Test" device on the outside of the ISS.

The moment of installation of the "Test" device by the astronaut is shown in Fig. 6.

After the exposure is completed, the astronaut places the test rods with cotton wool in a sterile transport module (1). Then this transport module is packed in a special delivery container.

### Ground-based research after exposure

After the end of the exposure period, the transport modules of the Test devices were delivered to the laboratory. Cotton wool with each strain of microorganisms was extracted from the modules separately in a sterile laminar box. Each cotton wool sample was transferred to the individual vial with saline solution. Vials with archaea were placed in an anaerobic environment.

#### Determination of the number of surviving microorganisms

To determine the number of surviving bacteria and fungi, bacteria were seeded on the surface of the nutrient medium of tripkase-soy agar, and fungi were seeded on the surface of PDA. The bacterial culture was incubated at 30 °C for 3 days, and the fungi were incubated for 7 days at 28 °C. The number of surviving culture was determined by the method of marginal dilutions. Then, different strains of bacteria and fungi were isolated from individual colonies based on the color change of the colony to create a collection and conduct subsequent experiments.

To determine the number of surviving archaea, they were seeded on the PBBM nutrient medium. The number of surviving archaea was determined by the method given above.

*SEM studies* of ultrathin archaea sections were carried out using the Reynolds method^16^. The cells were centrifuged, then fixed with a 1.5% solution of glutaraldehyde in 0.5 M of a cacodilate buffer (pH 7.2) at 4 °C for 1 h. Then the cells were washed three times with the same buffer and fixed with 1% OsO4 solution on 0.05 M cacodilate buffer (pH 7.2) for 3 h at 20 °C. The preparation was dehydrated in a series of alcohol solutions and poured into Epon 812 resin, fixed and stained first with a 3% solution of uranyl acetate in 70% ethanol for 30 min, and then with lead citrate. The sections were examined using a JEOL JEM-100B transmission electron microscope. The methane content was determined using a Pye Unicam 304 gas chromatograph (Great Britain) with a flame ionization detector. For the determination, a glass column (length 1 m, inner diameter 2 mm) filled with a Porapak Q, 80–100 mesh (Fluka, Germany) was used. The temperature of the column, injector and detector were 90 °C, 150 °C and 180 °C, respectively. The carrier gas was nitrogen with a flow rate of 20 ml/min.

#### Assessment of the radiation dose acquired by the samples during exposure

The absorbed radiation dose was calculated according to the method described in Ref.^39^. The calculation took into account the total effect of electrons and protons of the Earth's radiation belts, protons, neutrons and heavy charged particles of solar cosmic rays arising during solar flares, taking into account the influence of the Earth's magnetosphere in the ISS orbit, protons and nuclei of galactic cosmic rays that freely penetrate into the ISS orbit.

#### Determination of radiation sensitivity of fungal strains spores

To determine the radiation sensitivity of fungi spores, the method of survival of microorganisms under the influence of different doses of γ—radiation was used. For this purpose, 40 ml of suspension of each strain fungus was prepared at the concentration of 10^6^ spores/ml. Then the suspension of each strain was poured into individual sterile tubes of 5 ml, of which one tube was left in the laboratory to determine the initial concentration of spores. One test tube of each strain was exposed to **γ—**radiation of the isotope ^60^Co at doses of 1 kGy, 2.5 kGy, 5 kGy, 10 kGy and 15 kGy at the GU-200 M installation (with an average absorbed dose rate of 2.5 kGy/h). These studies were carried out in a seven-fold repetition. The absorbed dose was monitored by dosimeters. After radiation exposure, the number of surviving viable units of each strain was determined by seeding and subsequent limit dilutions of their suspension on the surface of the PDA nutrient medium according to the method described above. The maximum suppressive γ—radiation dose was determined by the growth or absence of it in the studied strains.

#### Determination of the reaction of fungal strains spores to the temperature stress factor

Suspensions of spores of strains with a concentration of 10^6^ CFU/ml were prepared for these studies. Tubes with 5 ml of suspension of each sample were heated to temperatures of 37 °C, 45 °C, 65 °C and 85 °C and kept at these temperatures for 15 min. The reaction was determined by the survival rate of fungal spores after this procedure. 1 ml of each suspension was poured into the wells created in the PDA nutrient medium and then were incubated for 7 days at 28 °C. Survival was assessed by the presence of growth of each strain.

#### Determination of the reaction of fungal strains spores to the effects of chemical stress factors

To determine the reaction, a uniform lawn was prepared by sowing each strain grown on the PDA nutrient medium in individual Petri dishes. For the studies, 1% and 3% hydrogen and the disinfectant solution Chlorapine (Petrospirt CJSC) with a chlorine content of 1% were used. Paper disks with a diameter of 8 mm were used for exposure. Each disk was impregnated with the appropriate solution and placed on the surface of a homogeneous lawn of the individual Petri dish of each strain under study. Then these Petri dishes were incubated at 28 °C for five days. After that, the size of the fungal growth zone was determined. All studies were carried out in three-fold repetition.

*The reliability (P)* was determined using single-factor analysis of variance^40^.

## Data Availability

Most of the data obtained or analyzed in the course of this study is included in this published article and is available from the relevant authors upon reasonable request.
